# Annual Meetings

**Published:** 1918-04-27

**Authors:** 


### Annual Meetings.

### Chelsea Hospital for Women.

The interest which the Royal Family take in the welfare and activities of the Chelsea Hospital for Women was referred to with pride by Mr. T. Dyer Edwardes, J.P., chairman of the Council, who presided last week, in the absence of the Marquess of Londonderry, at the forty-seventh annual general meeting of governors. Queen Mary, he said, had graciously attended the matinée held last month at the Palladium to assist in clearing off the debt of £14,000 on the new hospital. As a result of that performance £3,200 was secured, including £1,000 from the trustees of the Zunz Bequest, who had already given £10,000 to the rebuilding fund. Both the King and Queen were present at a Palladium matinée held in aid of the funds just four years ago. Further, Her Majesty had recently paid an informal visit to the hospital. Such marks of Royal favour were especially gratifying to the governors. The War Office had readily accepted an offer of the Council to place the first floor of the institution at the disposal of wounded officers. Captain T. G. Sorby, who for some time occupied a bed in the hospital, had generously given £200 towards the installation of a much needed *x*-ray apparatus. Annual subscriptions had increased during 1917 from £1,438 to £1,631. Last year 368 major operations and 248 minor ones had been performed.

### Queen Charlotte's Lying-in Hospital.

In the absence of Lord Portman and Sir Samuel Scott, Mr. F. W. Hunt, deputy-chairman of the hospital, presided last week at the annual court of governors. Moving the adoption of the report for 1917, he said that, since the beginning of the war, the committee had done all it could to help the wives of those who were fighting for their country. The total number of such mothers cared for had exceeded 5,000. During the past year 439 wives of sailors and soldiers were admitted to the institution, while 506 were attended in their own homes. In addition, wives of members of the Colonial Forces and of Belgians as well as of other refugees had been assisted. The hospital had been crowded beyond its capacity. A welcome addition to its funds had been received in the shape of a grant amounting to £1,500 from the Local Government Board. The grant was made under the Maternity and Child Welfare Scheme of the Board, in recognition of the excellent work which the hospital had been doing in its ante-natal and district midwifery departments. Donations, it was stated, had increased during the year under review.

### Belgrave Hospital for Children.

Although at the close of last year the hospital was in a position to bring forward a balance of £300, it was stated last week, at a meeting held under the presidency of the Earl of Plymouth, that, at the end of March, the institution had unfortunately found itself in debt to the extent of £840. An earnest appeal was made on behalf of the funds. The hospital was doing useful and important work for the young, inasmuch as during the quarter ended March 31 last 136 in-patients—84 medical and 52 surgical—had been treated, nine of them being under one year of age. Out-patients to the number of 1,923—993 medical and 930 surgical—had received attention during the same period, while 416 casualties had been dealt with. The total attendances of the out-patients amounted to 8,407.

